# Orbital- and millennial-scale Antarctic Circumpolar Current variability in Drake Passage over the past 140,000 years

**DOI:** 10.1038/s41467-021-24264-9

**Published:** 2021-06-24

**Authors:** Shuzhuang Wu, Lester Lembke-Jene, Frank Lamy, Helge W. Arz, Norbert Nowaczyk, Wenshen Xiao, Xu Zhang, H. Christian Hass, Jürgen Titschack, Xufeng Zheng, Jiabo Liu, Levin Dumm, Bernhard Diekmann, Dirk Nürnberg, Ralf Tiedemann, Gerhard Kuhn

**Affiliations:** 1 Alfred-Wegener-Institut Helmholtz-Zentrum für Meeres- und Polarforschung, Bremerhaven, 27568 Germany; 2 Leibniz Institute for Baltic Sea Research, Warnemünde,, 18119 Rostock Germany; 3 Helmoltz Centre Potsdam GFZ German Research Centre for Geosciences, Potsdam, 14473 Germany; 4 State Key Laboratory of Marine Geology, Tongji University, Shanghai, 200092 China; 5 Key Laboratory of Western China’s Environmental Systems, (Ministry of Education), College of Earth and Environmental Sciences, Lanzhou University, Lanzhou, 730000 China; 6 State Key Laboratory of Tibetan Plateau Earth System, Resources and Environment (TPESRE), Institute of Tibetan Plateau Research, Chinese Academy of Sciences, Beijing, 100101 China; 7 Alfred-Wegener-Institut Helmholtz-Zentrum für Meeres- und Polarforschung, Sylt, 25980 Germany; 8 MARUM–Center for Marine Environmental Sciences, University of Bremen, Bremen, 28359 Germany; 9 Senckenberg am Meer, Marine Research Department, Wilhelmshaven, 26382 Germany; 10 State Key Laboratory of Marine Resources Utilization in South China Sea, Hainan University, Haikou, 570228 China; 11 Southern University of Science and Technology, Department of Ocean Science and Engineering, Shenzhen, 518055 China; 12 Alfred-Wegener-Institut Helmholtz-Zentrum für Meeres- und Polarforschung, Potsdam, 14473 Germany; 13 GEOMAR, Helmholtz Centre for Ocean Research Kiel, Kiel, 24148 Germany

**Keywords:** Palaeoceanography, Palaeoclimate

## Abstract

The Antarctic Circumpolar Current (ACC) plays a crucial role in global ocean circulation by fostering deep-water upwelling and formation of new water masses. On geological time-scales, ACC variations are poorly constrained beyond the last glacial. Here, we reconstruct changes in ACC strength in the central Drake Passage in vicinity of the modern Polar Front over a complete glacial-interglacial cycle (i.e., the past 140,000 years), based on sediment grain-size and geochemical characteristics. We found significant glacial-interglacial changes of ACC flow speed, with weakened current strength during glacials and a stronger circulation in interglacials. Superimposed on these orbital-scale changes are high-amplitude millennial-scale fluctuations, with ACC strength maxima correlating with diatom-based Antarctic winter sea-ice minima, particularly during full glacial conditions. We infer that the ACC is closely linked to Southern Hemisphere millennial-scale climate oscillations, amplified through Antarctic sea ice extent changes. These strong ACC variations modulated Pacific-Atlantic water exchange via the “cold water route” and potentially affected the Atlantic Meridional Overturning Circulation and marine carbon storage.

## Abstract (web summary)

How the Antarctic Circumpolar Current (ACC) changed on glacial-interglacial time scales is not well known. Here, the authors present a 140,000 year long sediment record from the Drake passage and show both glacial-interglacial as well as millennial-scale variability which are linked to Atlantic variability and marine carbon storage.

## Introduction

The Antarctic Circumpolar Current (ACC) is the largest oceanic current system on Earth^1^. It represents the central distributor of globally important water masses, as it is intimately linked to the meridional overturning circulation cells of the adjoining Atlantic, Indian, and Pacific oceans. It connects wind-driven surface circulation and thermohaline deep and bottom water circulation with impacts on global salinity, nutrients, dissolved gasses, and heat transport^1,2^. The configuration of the frontal systems of the ACC with upwelling and downwelling cells is crucial for Antarctic climate, the modes of biological productivity, and the physical and biological carbon pump^3,4^. Understanding the magnitude and sensitivity of the ACC during the geological past is fundamental for assessing its role in the global Meridional Overturning Circulation (MOC), in particular with regard to ongoing and future anthropogenic climate change.

Restricted regional proxy evidence for changes in ACC flow speed on glacial-interglacial timescales has yielded heterogeneous results in the different sectors of the Southern Ocean^5–12^ (Supplementary Fig. 9). For example, most of the available subantarctic records from the Indian Ocean sectors^9,11^ of the ACC reveal enhanced glacial current speeds, which is contrasted by flow speed reconstructions in the subantarctic eastern South Pacific^8^ and the subantarctic Atlantic sector^13^ with decreased flow (Supplementary Fig. 9). High-resolution reconstructions of the ACC flow speed at the entrance of Drake Passage (MD07-3128) and on its northern continental slope (MR0806-PC09) extend only back to ~65 ka but show substantial glacial decreases^6^. In contrast, time-slice reconstructions from the Scotia Sea downstream of the Drake Passage show only small flow speed changes for the Last Glacial Maximum (LGM) compared to the Holocene^5^. Knowledge for the ACC changes in the central Drake Passage beyond the last glacial cycle is still unclear. Furthermore, underlying forcing mechanisms remain largely elusive^5–8^. At present, the ACC strength is mainly driven by the southern westerly winds (SWW) and surface buoyancy forcing^14,15^. Previous studies proposed that sea ice cover, in addition to wind stress, has a significant influence on the surface ocean drag coefficient on forcing the ocean surface layer and ultimately current speed^5,6,16^.

**Fig. 1:**
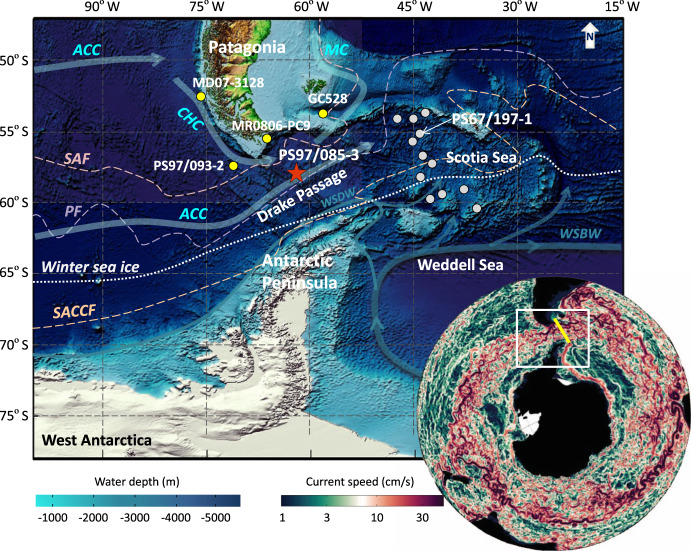
Location map. Core PS97/085-3 is located in the central Drake Passage (red star), ~40 km north of the Polar Front (see Supplementary Fig. 1). Yellow dots mark sediment cores north of the Subantarctic Front^6–8^. Gray dots indicate Scotia Sea transect cores south of the Polar Front^5^. Core PS67/197-1 in the Scotia Sea is sensitive to changes in winter sea ice extent^22^. Light blue arrows show the Antarctic Circumpolar Current (ACC), the Cape Horn Current (CHC) and Malvinas Current (MC)^64^, while dark green-blue arrows denote Weddell Sea Bottom Water (WSBW, thick) and Weddell Sea Deep Water (WSDW, thin) flows. The white dotted line marks the average modern winter sea ice edge^68^. Dashed lines are the Subantarctic Front (SAF, pink), Polar Front (PF, purple) and the Southern Antarctic Circumpolar Current Front (SACCF, orange)^17^. The right bottom insert map is modified from Rintoul. (2018)^2^ shows current speed in the Southern Ocean, with warmer red colors representing higher current speeds. The white box shows the study area and the yellow line marks the Jason Track 104^36^. The figure is adapted from Wu et al.^26^.

The Drake Passage is the major “bottleneck” (Fig. 1) along the eastward ACC path around Antarctica, subdivided by three principle oceanographic fronts: the Subantarctic Front (SAF), the Polar Front (PF), and the Southern ACC Front (SACCF)^17^. Subsidiary fronts were further determined by satellite altimetry showing multiple jets along the ACC pathway^18^. The ACC transports cold and fresh water from the Pacific to the Atlantic through the Drake Passage, known as “cold water route”^19^. This cold water route complements the warm westward Agulhas Current or “leakage” off the Cape of Good Hope known as “warm water route”. These two sources, together with deep waters originating from the Southern Ocean, comprise the northward-flowing return path balancing the southward migration of the Circumpolar Deep Water component of the Atlantic Meridional Overturning Circulation (AMOC)^1,2,19–21^.

In this work, we reconstruct changes in Pacific-Atlantic ACC flow speed on millennial to glacial-interglacial time scales over the past 140 ka. We used high-resolution grain-size data of the siliciclastic sediment fraction and X-ray fluorescence (XRF) scanning-based elemental ratios from a sediment core in the central Drake Passage (Site PS97/085; 58° 21.28′S, 62° 10.02′W; 3090 m water depth; Fig. 1) as bottom flow speed proxies. The age model is based on a combination of paleomagnetic excursions, relative paleointensity (RPI), radiocarbon dates, and tuning of high-resolution XRF scanning-derived calcium to titanium ratios (ln(Ca/Ti)) to Antarctic temperature anomalies (see Supplementary Methods; Supplementary Table 1). Linear sedimentation rates range between ~2 and 40 cm/ka, with higher mass accumulation rates during full glacial periods (Supplementary Fig. 3). In addition, we extend a previously published diatom-based winter sea ice record from the Scotia Sea further back in time to 60 ka (Site PS67/197, 55° 8.24′S, 44° 6.28′W; 3837 m water depth; Fig. 1)^22^. The chronology of this latter core has been published before and is used here without modification^23^. Combining our ACC flow speed proxies with sea-ice reconstructions enables us to investigate millennial-scale ACC variations, potentially linked to Antarctic winter sea ice changes during the last glacial period.

## Results and discussion

### ACC flow speed proxies for the Drake Passage

**Fig. 2:**
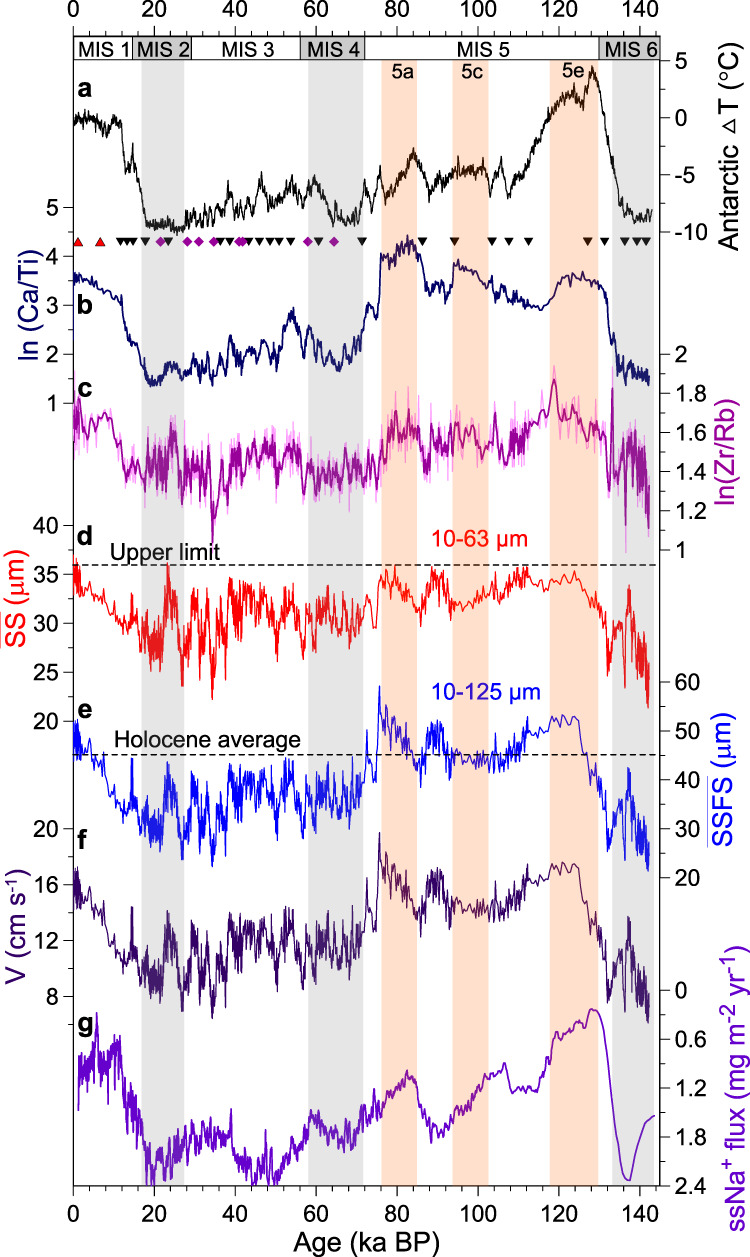
Reconstructed changes in ACC strength and compared with Antarctic temperature and Southern Ocean sea-ice extent. **a** Antarctic temperature changes from the European Project for Ice Coring in Antarctica (EPICA) Dome C ice core^47^. **b** High-resolution XRF scanner-derived records of ln(Ca/Ti) (peak area count ratios; black triangles) were applied to fine-tune the Antarctic temperature anomalies together with radiocarbon dates (red triangles), relative paleointensity and paleomagnetic excursions (purple diamonds) age control points from core PS97/085-3 (see Supplementary Methods, Supplementary Figs. 2 and 3). **c** XRF-derived ln(Zr/Rb) variations indicate changes in sediment grain-size fractions. **d** Mean sortable silt grain size ($\overline{{{{\rm{SS}}}}}$, 10–63 μm) reaches up its upper limit under high flow speeds. **e** Mean grain size of sortable silt and fine sand ($\overline{{{{\rm{SSFS}}}}}$, 10–125 μm) was used as the ACC flow speed proxy in this study. **f** ACC bottom flow speeds were estimated by the correlation between the $\overline{{{{\rm{SSFS}}}}}$ and adjacent current meter data (see Supplementary Fig. 6). **g** Sea salt sodium (ssNa^+^) flux from the EPICA Dronning Maud Land (EDML) ice core, a proxy for sea ice extent and atmosphere changes, smoothed with a three-points running mean^34^. Vertical gray bars mark inferred glacial periods and pink bars indicate the sub-interglacial during Marine Isotope Stage (MIS) 5.

We use changes in grain size and geochemical properties of the terrigenous sediment fraction to reconstruct changes in ACC strength (Fig. 2). The linear function between sortable silt mean grain sizes ($\overline{{{{\rm{SS}}}}}$, 10–63 μm) and scalar bottom current velocities is typically applied for evaluating relative changes in the near-bottom velocities in deep-sea sediments^24,25^. We correlated near-bottom velocities mooring measurements with $\overline{{{{\rm{SS}}}}}$ of seafloor sediments on a north-south Drake Passage transect^26^. However, modern observations^27^ reveal that ACC flow speeds at ~3000 m water depth can reach up 40–60 cm s^−1^; such high flow speeds at the sea floor can potentially remove parts of the silt and even the sand fractions^24^. The grain-size distribution mode would thus shift to the coarser fractions dominated by fine sand (Supplementary Fig. 4, see Supplementary Methods), and hence impair the $\overline{{{{\rm{SS}}}}}$ as a current speed proxy. Accordingly, the $\overline{{{{\rm{SS}}}}}$ may not capture the entire magnitude of the flow-speed variations in our record (Fig. 2d, see Supplementary Methods). High ACC speed can extend the sorting range beyond the sand-silt boundary (Supplementary Fig. 4), which was also observed at the Chilean Margin and the northernmost Drake Passage^6^ (Supplementary Fig. 8). Therefore, we use the mean grain size of the sortable silt plus the fine sand fractions ($\overline{{{{\rm{SSFS}}}}}$, 10–125 μm) as flow-speed proxy to reconstruct deep ACC dynamics throughout the past 140 ka (Fig. 2e). Since our site is ~400 km away from the South American continent and Antarctica, the terrigenous sand fractions are most likely transported by various processes (including sea ice, icebergs, etc.) from the continents and then reworked by bottom currents. Generally, unsorted ice-rafted debris (IRD) might affect the $\overline{{{{\rm{SS}}}}}$-based flow speed proxy^28^. However, the content of IRD in our sediment record is generally less than 2 vol.% and 25 clasts cm^−3^ (Supplementary Fig. 5). One spike with ~18 vol.% at the end of Marine Isotope Stage (MIS) 4 might overestimate large clasts (see Supplementary Methods). IRD fluctuations are overwhelmingly independent of $\overline{{{{\rm{SSFS}}}}}$ changes with short-term exceptions at 22–24 ka and in the oldest part beyond ~134 ka (Supplementary Fig. 5). Furthermore, variations in $\overline{{{{\rm{SSFS}}}}}$ and SSFS% component are positively correlated (*r*^2^ = 0.8; Supplementary Fig. 5), suggesting bottom currents are the principal driver for changes in the grain-size fraction (10–125 μm) at our site^24,28^.

Zirconium (Zr) is typically accumulating in the heavy mineral fraction associated with the sand fractions, especially fine sand (63–125 µm), while rubidium (Rb) is preferentially retained in the clay mineral faction^29^. Previous studies found a positive correlation between grain size and the Zr/Rb ratio^30,31^. In case, where the depositional regime is dominated by current sorting, the ln(Zr/Rb) ratio can indicate bottom current strength^31^. Our record shows a positive correlation between $\overline{{{{\rm{SSFS}}}}}$ and ln(Zr/Rb) ratios (*r*^2^ = 0.6, *n* = 1480). Major aeolian input can be ruled out in our study area by clay mineralogy and geochemical properties^26,32,33^. High-resolution records of grain size and Zr/Rb ratios can thus provide a robust signature of ACC variability.

### Changes in ACC strength in the central Drake Passage

**Fig. 3:**
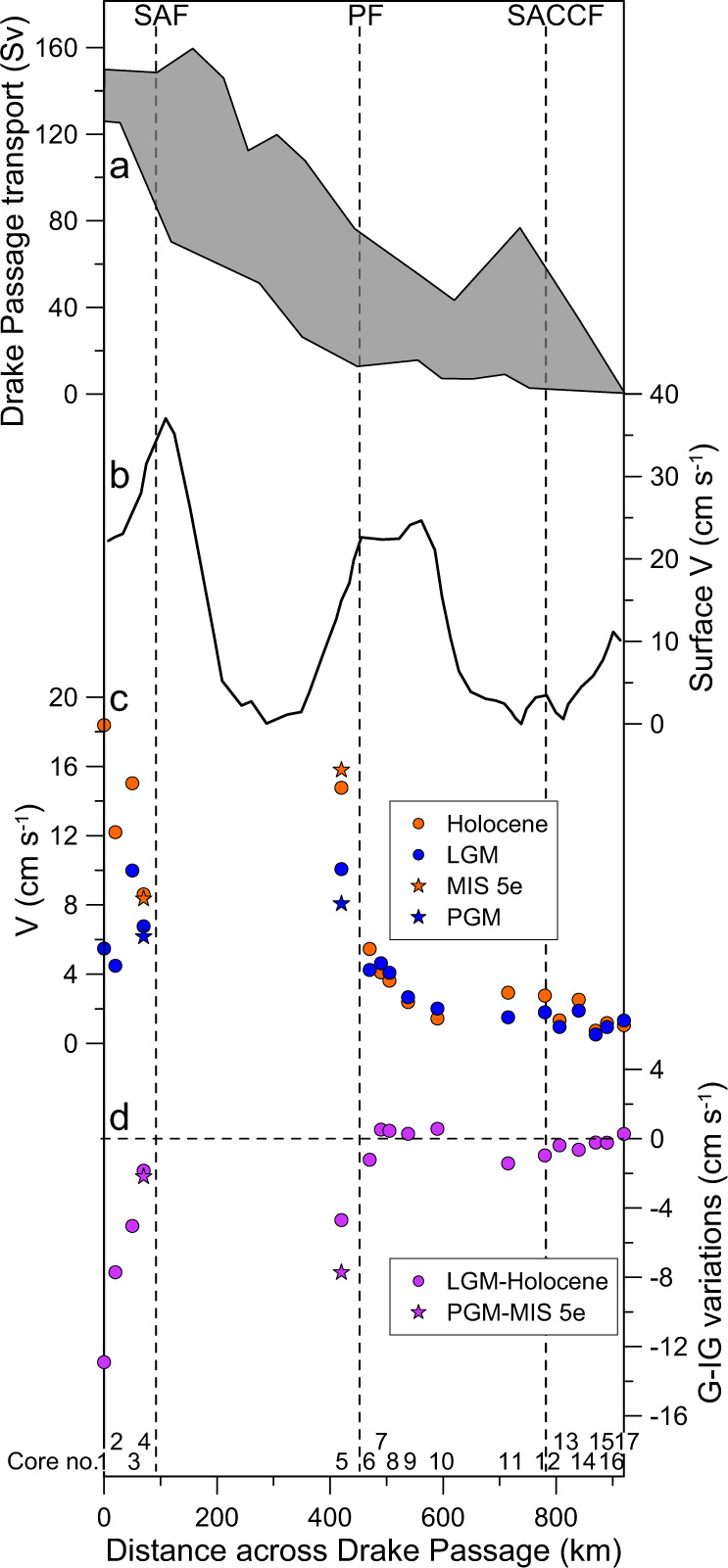
Comparison of the ACC flow speeds in the Drake Passage region. **a** Range of modern Drake Passage cumulative volume transport from south to north along Jason Track 104^36^ and SR1b^69^. **b** Mean cross-track surface geostrophic velocities^36^. **c** Bottom flow speeds are transferred by the latest calibration in the Drake Passage region^26^ and the new $\overline{{{{\rm{SSFS}}}}}$ flow speed proxy explained in Supplementary Fig. 6. Orange and blue dots mark ACC flow speeds during the Holocene and LGM^5–8^, respectively. Orange and blue stars indicate flow speed in the Drake Passage during the MIS 5e and PGM, respectively. **d** Glacial-interglacial (G-IG) flow speed differences associated with frontal system. Larger glacial reductions of flow velocities are detected in north of the SAF (cores 1-4, see Table S2)^6–8^ and the PF (core 5, this study) compared to small changes south of the PF (cores 6–17;^5^ see Supplementary Table 2). SAF Subantarctic Front, PF Polar Front, SACCF southern ACC Front.

Our sediment record reveals glacial-interglacial variations in ACC flow speed in the central Drake Passage over the past 140 ka (Fig. 2). The timing of major ACC changes follows Antarctic temperature variations^34^, implying the ACC strength in the Drake Passage is sensitive to large-scale Southern Hemisphere climate oscillations (Fig. 2a, c, e). The last interglacial (MIS 5e, ~129–116 ka) was warmer than today^35^ and our record indicates average $\overline{{{{\rm{SSFS}}}}}$ of ~48 μm. During the stable part of MIS 5e (~123–118 ka), our record shows a plateau structure with average $\overline{{{{\rm{SSFS}}}}}$ of ~52 μm, which is higher than the Holocene average (~0–12 ka, ~45 μm) within measurement uncertainty (Fig. 2e). This suggests that the ACC flow speed was enhanced during MIS 5e, supported by corresponding changes in ln(Zr/Rb) ratios during the stable MIS 5e (~1.73) and the Holocene (~1.65) (Fig. 2c). In contrast to MIS 5e and the Holocene, the ACC strength significantly decreased during the Penultimate Glacial Maximum (PGM, ~140 ka; $\overline{{{{\rm{SSFS}}}}}$ = ~27 μm) and the LGM *sensu lato* (~28–18 ka; $\overline{{{{\rm{SSFS}}}}}$  = ~32 μm). Given the correlation between the $\overline{{{{\rm{SSFS}}}}}$ and adjacent current meter, the calculated mean bottom current speeds vary from ~6 to 20 cm s^−1^ in the center of the Drake Passage during the last glacial cycle (Fig. 2f and Supplementary Fig. 6). The record shows a ~8 cm s^−1^ increase in the ACC from the PGM to MIS 5e, larger than the LGM to Holocene increase of ~5 cm s^−1^. High-frequency variability in millennial-scale ACC changes prevailed during the glacial periods, but the ACC flow speed maxima always remained below ~15 cm s^−1^ during this interval (Fig. 2f). Noteworthily, the calculated flow speeds are depending on the sensitivity of the $\overline{{{{\rm{SSFS}}}}}$ to the ACC flow speed that largely may rely on local conditions like bathymetry and seafloor morphology. Standard deviation of modern hourly current measurements is 8.2 cm s^−1^ at Site C18^27^ which is similar to the PGM-MIS 5e amplitude (Fig. 3). Therefore, large uncertainties of about 50% of the calculated values thus exist owing as well to sparsely long-term measured data (see Supplementary Methods). A more reliable quantification of the ACC in the Drake Passage would require long-term current meter records, which are presently not available, and at sites where grain-size measurements exist or will be studied.

Here, we combine our records with published grain size data^5–8^ transferred by the latest calibration^26^ to explore glacial-interglacial changes in ACC strength associated with oceanic fronts in the Drake Passage region (Fig. 3; Supplementary Table 2). Larger glacial-interglacial changes in flow speeds were observed north of the SAF with a range of ~5–13 cm s^−1^ (MD07-3128, MR0806-PC9, and GC528) at intermediate water depths (~600–1030 m)^6,7^. Smaller fluctuations in ACC changes (~2 cm s^−1^, PS97/093-2) occurred in deep water (~3781 m) near the SAF^8^. In the central Drake Passage ~40 km north of the PF, the ACC flow speeds exhibit a ~5–8 cm s^−1^ glacial reduction at ~3090 m water depth, which is considerably greater than variations along the Scotia Sea transect south of the PF (< 2 cm s^−1^, Scotia Sea transect cores, ~2000–4300 m water depth)^5^. Differences between these glacial-interglacial changes are likely due to the geographical settings within latitudinal subdivisions of the ACC and the related fronts (Fig. 3). Therefore, we suggest an enhanced sensitivity of the ACC to glacial-interglacial climate changes around the PF and the SAF^6–8^, in contrast to minimal changes south of the PF along the Scotia Sea transect^5^. Such presumption would be consistent with modern ACC volume transport and highest current velocities prevailing within the multiple jets of the SAF and PF in the Drake Passage region^18,27,36^.

Several processes might cause these changes in the ACC strength at site PS97/085, including changes in the strength and latitudinal position of SWW^14^, oceanic frontal shifts^37^, and buoyancy forcing^15^. Although the intensity and position of the glacial SWW remain uncertain, a northward displacement of the SWW is widely assumed during the LGM^38,39^, with reduced impact on the ACC in the Drake Passage^5,6,8^. The glacial oceanic fronts were likewise suggested to have shifted equatorward^37^, thus the South American continent would have obstructed the ACC flow through the Drake Passage^7^. Moreover, changes in ACC strength on the glacial-interglacial timescale correspond to the sea salt sodium (ssNa) flux record from the East Antarctic Dronning Maud Land (EDML) ice core (Fig. 2e–g), a proxy partially related to large-scale sea ice production^34^. This indicates that major changes in circum-Antarctic sea ice cover might have been linked to glacial-interglacial ACC strength changes, in line with earlier suggestions^5,6^. During the LGM, summer sea ice extent likely expanded northward by at least ~500-km^37,40^. Such large sea ice coverage could have significantly decreased the efficiency of wind stress acting on the ocean surface^5,6,16^ and thus reduced the ACC strength (Figs. 2f and 3c). Conversely, at interglacial stages, sea ice retreat and southward displacement of the SWW and oceanic fronts would have increased wind stress efficiency on the ocean surface and accelerated the ACC speed^5,6,22^. However, the sub-orbital ACC changes in our record are not evident in the ssNa flux pattern, e.g., during late MIS 5, MIS 3 and 2 (Fig. 2e–g). We speculate that the ssNa flux in the EDML ice core may not have captured short-term regional changes in sea ice extent, but was influenced by changes in the hydrologic cycle as well^34,41^.

### Millennial-scale variations of ACC during the last glacial

**Fig. 4:**
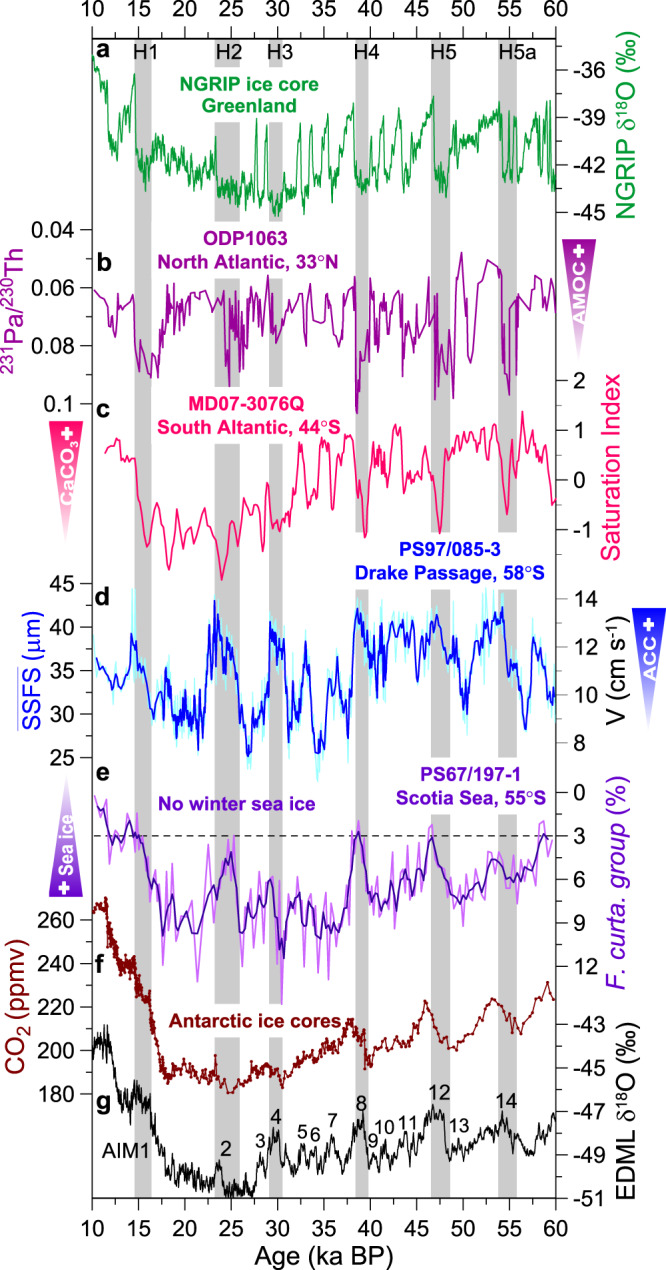
Millennium-scale changes in ACC strength compared to paleoclimatic records over the last 60 ka. **a** Oxygen isotope record from North Greenland Ice Core Project (NGRIP, δ^18^O vs. VSMOW)^70^. **b** Bermuda Rise ^231^Pa/^230^Th proxy for the AMOC strength^44,59,60^. **c** Saturation index as a proxy for reconstructed carbonate saturation changes in the South Atlantic^49^. **d** $\overline{{{{\rm{SSFS}}}}}$ as flow speed proxies for the current strength (three-point smoothing, this study) and the transferred ACC flow speeds. **e** Relative abundance of diatom winter sea ice indicator *Fragilariopsis curta* group (*F. curta* + *F. cylindrus*) with three-point smoothing^22,67^. The group abundance >3% indicates the presence of winter sea ice^22,67^. **f** Synchronized ice-core atmospheric *p*CO_2_^45^. **g** δ^18^O time series from the EDML ice core^45^. Vertical gray bars mark inferred millennial-scale ACC peaks that correspond with millennial-scale temperature maxima in Antarctica (AIM) and Heinrich events (H) in Greenland.

Superimposed on the glacial-interglacial changes, our records exhibit marked high-amplitude, millennial-scale variations in the ACC flow speeds covering the last glacial cycle with highest amplitudes between MIS 4 and MIS 2 (Fig. 4d). Overall, a stronger ACC coincides with a weakened AMOC during cold phases in the Northern Hemisphere and warm intervals in the Southern Hemisphere (Fig. 4a, b, d). This pattern is consistent with the bipolar temperature seesaw and a strong atmospheric link between the North Atlantic realm across the tropics into the SWW system and subsequently also the ACC^42,43^. For the last glacial termination, the AMOC shutdown^44^ during Heinrich Event 1 reinforced the insolation-driven initial Southern Hemisphere deglacial warming^45^. Together with the previous records^6^, our data indicate that the ACC might play an important role in atmosphere-ocean changes and the interplay between millennial- and orbital-scale changes during glacial terminations. Furthermore, we found increasing amplitudes of ACC millennial-scale variations as the climate was approaching the LGM (~38–23 ka; Fig. 4d), in contrast to a steady decline of the Antarctic temperatures (Fig. 4g). Enhanced sensitivity of the ACC towards the LGM was also recorded in the variation of the fine sand fraction percentages in the northern ACC/Cape Horn Current^6^ (Supplementary Fig. 8). This suggests the ACC reached its highest sensitivity to Southern Hemisphere millennial-scale climate oscillations during full glacial conditions, with the ACC re-accelerating to higher flow speeds during Antarctic warming events associated with Northern Hemisphere Heinrich Stadials^46^.

Supporting evidence for causes of millennial-scale ACC strength changes near our site is scarce. While regional temperature proxy records from Antarctica^47^ and off southernmost South America^7,48^ show some correspondence to our ACC strength reconstruction, remaining dissimilarities indicate that factors other than temperature changes played a role in forcing ACC flow variations. Previous ACC strength reconstructions from sites north of our location attributed variations to SWW^6^ changes associated northward shifts of the SAF^7^. However, model simulations of millennial-scale SWW changes indicate relatively small-scale variations during glacial periods^49^, which alone may have been insufficient to drive high-amplitude changes in ACC speeds from surface to 3090 m water depth (Fig. 4d). We thus chose to investigate potential regional amplification effects of sea ice changes on millennial-scale ACC dynamics, as the EDML ssNa flux did not indicate hemispheric-wide sea ice variations^34^ on these time-scales.

We reconstructed winter sea ice extent from diatom assemblages in a temporally well-resolved sediment record from the northern Scotia Sea, downstream of Drake Passage (PS69/197-1, Fig. 4e). Within age uncertainties, we observe a close correspondence between millennial-scale maxima in ACC strength and major winter sea ice retreat intervals (Fig. 4d, e). Such sizeable variability of seasonal sea ice is also documented in other diatom records from the Scotia Sea, which revealed seasonal sea ice shifts by up to ~8° latitude that have been attributed to changes in regional sea surface temperatures, and austral summer insolation forcing^50^. Likewise, under modern conditions winds play a major control on the extent of sea-ice^51^. We propose that Antarctic sea ice extent, together with wind changes, acted as a positive feedback mechanism on increasing amplitudes in millennial-scale ACC strength towards the LGM. Sea ice effectively moderates the momentum transfer of wind-derived energy into the surface ocean^5,16^, because the air drag over ice and open water fundamentally differs and scales with the density of sea ice cover. The effect of sea ice on the flow depends on the ratio of ice floes to patches of open water^5,16,52^. In the dynamic Drake Passage, a highly mobile sea ice cover with an optimal concentration (50–90%) would constitute a strong catalyst between wind and the surface ocean^16,52^. Such optimal sea ice conditions could increase the air-sea drag coefficient by a factor of two to four^16,52^, thus yield a strong amplification of glacial millennial-scale variations in SWW forcing on the ocean surface. We hypothesize that increasing amplitudes in millennial-scale ACC flow speed variations approaching the LGM have been likely linked to variations of Antarctic sea ice extent.

Changes in ACC strength could have regulated the Pacific-Atlantic exchange and caused variations in the chemical ventilation of the Drake Passage and South Atlantic water masses. On glacial-interglacial timescales, a weaker ACC in combination with a more extensive sea ice cover likely resulted in more sluggish deep waters and a stratified glacial circulation mode with a more isolated lower cell as detected by specific Nd-isotope values in deep-sea corals^53^ and Pb- and Nd-isotopic composition of authigenic Fe-Mn oxyhydroxide sediment coatings^54^. A comparison of our ACC strength record to a high resolution carbonate saturation reconstruction from the Subantarctic South Atlantic^49^ indicates a correspondence of stronger ACC with reduced carbonate saturation during the major Antarctic warm intervals (Fig. 4b–d). These changes in inflow of Pacific-type deep-water masses likely influenced the carbonate chemistry in the subantarctic South Atlantic competing with North Atlantic sourced-deep waters at millennial time-scales^49,55^. The contribution of Antarctic Bottom Water circulation to the modern ACC is modest, but potentially increased during glacial periods when expanded sea ice favored its production and increased its salinity^12,40^. In combination with wind-driven upwelling^42^ and changes in sea-ice extent^40,53,56^ these Southern Ocean processes modulate sequestered CO_2_ exchange (Fig. 4f) with the atmosphere over millennial timescales.

### Physical changes in the ACC linked to the AMOC stability

**Fig. 5:**
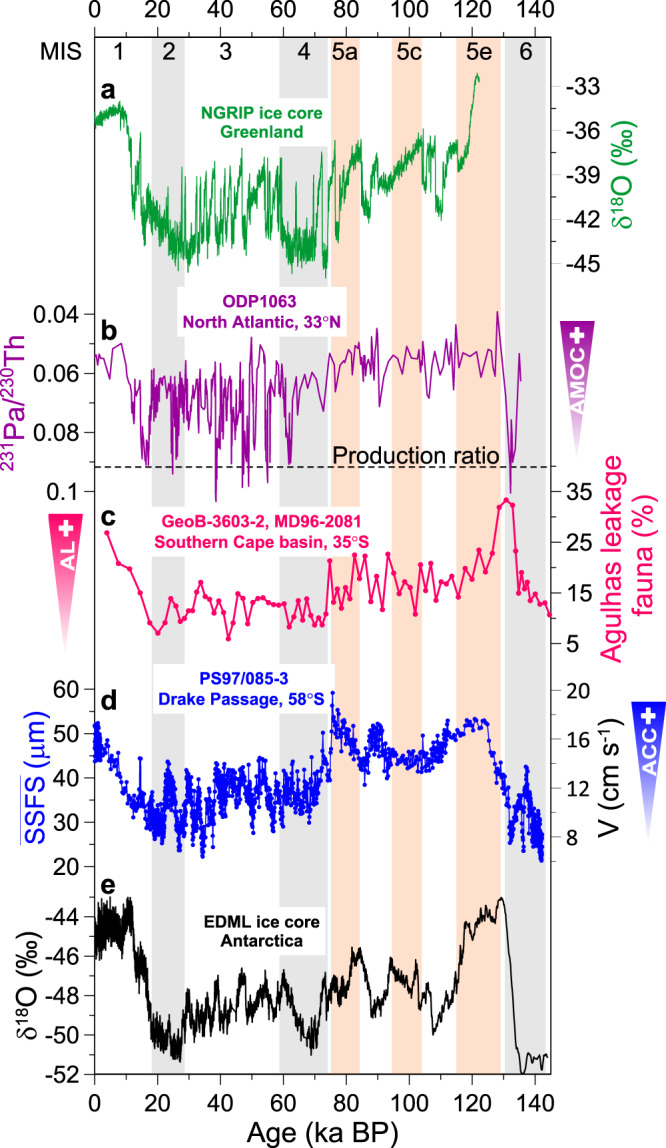
Reconstructed changes in ACC strength compared with paleoclimatic records over the past 140 ka. **a** NGRIP oxygen isotope record^70^. **b** Bermuda Rise ^231^Pa/^230^Th data^44,59,60^, indicating the AMOC strength. Dashed line is production ratio (^231^Pa/^230^Th = 0.093), suggesting no transport. **c** Planktic foraminiferal fauna reflect the intensity of the Agulhas leakage^58^. **d** $\overline{{{{\rm{SSFS}}}}}$ as a flow-speed proxy for the current strength (blue, this study) and the transferred ACC flow speeds. **e** Oxygen isotope record from the EDML ice core^45^. Vertical gray bars mark glacial periods and pink bars mark the warm stages during MIS 5.

Changes in ACC transport through the cold water route, together with the Agulhas leakage via the warm water route, have been suggested to regulate the AMOC strength^6,20,21^. However, the relative contribution of these two water routes for the upper branch of AMOC has rarely been addressed in the past^20,57^. We compared our Drake Passage throughflow strength (Fig. 5d) with the Agulhas leakage intensity, reconstructed from planktic foraminiferal fauna census counts^58^ in the South Africa margin over the past 140 ka (Fig. 5c). Both water routes’ transport increased during the past two deglaciations^58^ (Fig. 5c, d), suggesting that they both likely have induced a positive feedback to the AMOC recovering into a stronger interglacial mode as indicated by low ^231^Pa/^230^Th ratios^44,59,60^ (Fig. 5b).

The Agulhas leakage reached its transient transport maximum during the terminations, with subsequently significantly decreased water volume transport to the South Atlantic (~8 Sv during MIS 5) during interglacials^61^. In contrast, the Drake Passage throughflow remained strong throughout MIS 5, with smaller sub-interglacial fluctuations (Fig. 5d). Extremes in total ACC volume transport were estimated over some weeks during a 20-year period with maximum 166 Sv and minimum 105 Sv and a mean of 140 Sv^62^. Reanalysis for a 25 years observation period provides mean total transport volume through Drake Passage of 155 ± 3 Sv^63^ and ~62% of subsurface water (50–1554 m, float depth) is flowing into the Malvinas Current^64^. Therefore, for our maximum velocity calculations during peak warm interglacials like MIS 5e we assume a total volume transport through Drake Passage of about 166 Sv. Relative glacial decrease (in % of interglacial values) of bottom water flow velocities show a mean reduction of 43 ± 19% (Fig. 3d, Supplementary Table 2) north of the PF. Therefore, we estimate an equivalent reduction for the ACC volume transport during glacial periods delivering 88 ± 29 Sv. The changes south of the PF are small and were not considered for this estimation (see Fig. 3d). Our record show higher ACC flow speeds during most of MIS 5 than the Holocene average (Fig. 5d). Such a strong ACC might have enhanced the formation of surface and intermediate water to fuel the upper overturning cell in the Southern Ocean during MIS 5. In contrast to the lower Agulhas leakage transport, the cold water route may have played a crucial role in keeping the AMOC vigorous throughout MIS 5. A slow-down of the ACC at the MIS 5/4 transition, followed by high frequency variations through MIS 4 to MIS 2, while a sluggish Agulhas leakage prevailed during the last glacial period^58^ (Fig. 5c, d). These strong variations of the ACC through the cold water route have been likely linked to high AMOC instability^46,48^ between MIS 4 and MIS 2 (Fig. 5b).

Compared to the Holocene, our data imply a stronger ACC prevailed during the last, warmer-than-present interglacial^35^. This would be consistent with an overall stronger ACC together with strengthened westerly winds under warming climate^2^. Under warmer-than-present conditions, such a persistent cold water route return flow into the Atlantic could stabilize the AMOC in the long-term future, despite the AMOC showing emerging signs of weakening over the past decades^65^ and recently is at its weakest stage during the last millennium^66^. Although the ACC provided a dynamical link with the AMOC, several other important processes, like Southern Ocean wind-driven upwelling and buoyancy forcing^2,14,42^, may act together to explain the full changes of the AMOC.

## Methods

### Paleomagnetic measurements

Volume susceptibility on core PS97/085-3 was performed with a Bartington MS2E sensor and MS2 control unit on a split-core logger in 1 mm intervals and a sensor amplitude resolution of 10^−5^ (SI-Units). The slope of Natural remanent magnetization intensity versus anhysteretic remanent intensity of common demagnetization steps was determined in order to provide a proxy for the relative paleointensity (RPI) (see Supplementary Methods).

### X-ray fluorescence core scanning (XRF-CS)

The sediment core was measured with an AVAATECH XRF-CS at the Alfred Wegener Institute (AWI), Bremerhaven. XRF-CS data were collected in 1 cm steps (area 10 × 12 mm) along the core in three runs with 10 kV, 30 kV, and 50 kV (see Supplementary Methods).

### Age model

The age model is based on a combination of radiocarbon dates, paleomagnetic excursion, correlation of RPI of core PS97/085-3 with the RPI stack and tuning from high resolution XRF-CS ln (Ca/Ti) (Supplementary Table 1). Bayesian age-depth modeling program Bacon 2.3 was applied to develop an age model based on radiocarbon dates, paleomagnetic and tuning points (Supplementary Fig. 2). The error estimate (Supplementary Table 1) for tuning points and paleomagnetic tie points was done using mean squared estimate (details and reference see Supplementary Methods).

### Grain-size measurements

A total of 1520 samples were taken in 1 cm intervals from core PS97/085-3 for grain-size measurements with a CILAS 1180L laser diffraction particle-size analyzer (CILAS, Orleans, France) at the AWI, Sylt. For comparison of different grain-size measurement methods, 80 samples were measured with a Micromeritics SediGraph 5100 at AWI, Bremerhaven (see Supplementary Methods and Supplementary Fig. 7).

### Diatom census

The sample preparation of permanent mounted slides from core PS67/197-1 for microscopic diatom census followed the standard procedure established at the AWI^67^. Diatom species and species groups were identified and counted with a minimum of 400 specimens, at the magnification of ×1000 using a Zeiss microscope. Relative abundances of sea ice related species *Fragilariopsis curta* and *Fragilariopsis cylindrus* were combined for estimation of changes in winter sea ice extent^67^ (Supplementary Fig. 8).

## Supplementary information

SUPPLEMENTARY INFO

## Data Availability

All relevant data in this paper are available at PANGAEA Data Publisher (https://doi.pangaea.de/10.1594/PANGAEA.923843).
