# Author Correction: Orbital- and millennial-scale Antarctic Circumpolar Current variability in Drake Passage over the past 140,000 years

**DOI:** 10.1038/s41467-021-27840-1

**Published:** 2022-01-10

**Authors:** Shuzhuang Wu, Lester Lembke-Jene, Frank Lamy, Helge W. Arz, Norbert Nowaczyk, Wenshen Xiao, Xu Zhang, H. Christian Hass, Jürgen Titschack, Xufeng Zheng, Jiabo Liu, Levin Dumm, Bernhard Diekmann, Dirk Nürnberg, Ralf Tiedemann, Gerhard Kuhn

**Affiliations:** 1 Alfred-Wegener-Institut Helmholtz-Zentrum für Meeres- und Polarforschung, Bremerhaven, 27568 Germany; 2 Leibniz Institute for Baltic Sea Research, Warnemünde,, 18119 Rostock Germany; 3 Helmoltz Centre Potsdam GFZ German Research Centre for Geosciences, Potsdam, 14473 Germany; 4 State Key Laboratory of Marine Geology, Tongji University, Shanghai, 200092 China; 5 Key Laboratory of Western China’s Environmental Systems, (Ministry of Education), College of Earth and Environmental Sciences, Lanzhou University, Lanzhou, 730000 China; 6 State Key Laboratory of Tibetan Plateau Earth System, Resources and Environment (TPESRE), Institute of Tibetan Plateau Research, Chinese Academy of Sciences, Beijing, 100101 China; 7 Alfred-Wegener-Institut Helmholtz-Zentrum für Meeres- und Polarforschung, Sylt, 25980 Germany; 8 MARUM–Center for Marine Environmental Sciences, University of Bremen, Bremen, 28359 Germany; 9 Senckenberg am Meer, Marine Research Department, Wilhelmshaven, 26382 Germany; 10 State Key Laboratory of Marine Resources Utilization in South China Sea, Hainan University, Haikou, 570228 China; 11 Southern University of Science and Technology, Department of Ocean Science and Engineering, Shenzhen, 518055 China; 12 Alfred-Wegener-Institut Helmholtz-Zentrum für Meeres- und Polarforschung, Potsdam, 14473 Germany; 13 GEOMAR, Helmholtz Centre for Ocean Research Kiel, Kiel, 24148 Germany

**Keywords:** Palaeoceanography, Palaeoclimate, Palaeoceanography, Palaeoclimate

Correction to: *Nature Communications* 10.1038/s41467-021-24264-9, published online 24 June 2021.

The original version of this Article contained an error in Fig. 1, in which the unit for the variable “Current speed” was given as “m/s”, but should be “cm/s”. This has been corrected in both the PDF and HTML versions of the Article.

