# Supplementary material for: Orbital- and millennial-scale Antarctic Circumpolar Current variability in Drake Passage over the past 140,000 years
Source: Nat Commun. 2021 Jun 24;12:3948. doi: 10.1038/s41467-021-24264-9 (PMC8225899; doi:10.1038/s41467-021-24264-9)
Supplement: Supplementary file 1 — SUPPLEMENTARY INFO [file 41467_2021_24264_MOESM1_ESM.pdf]

Supporting Information for "Orbital- and millennial-scale Antarctic Circumpolar Current variability in Drake Passage over the past 140,000 years"

S. Wu*, L. Lembke-Jene, F. Lamy, H.W. Arz, N. Nowaczyk, W. Xiao, X. Zhang, H. C. Hass, J. Titschack, X. Zheng, J. Liu, L. Dumm, B. Diekmann, D. Nürnberg, R.Tiedemann & G. Kuhn

Material and methods

Sediment composition. During Polarstern Expedition PS97, piston core PS97/085-3 (and corresponding trigger core) were retrieved from the central Drake Passage at 58° 21.27′S, 62°10.03′W (water depth 3090 m; 14.43 m core length; trigger corer 0.89 m core length)[1]. Our site PS97/085 is located in the vicinity of the West Scotia Ridge spreading center and lies on a crest of the northern flank of the West Scotia Ridge next to the Shackleton Fracture Zone (Supplementary Fig. 1). The piston and trigger corers were spliced together by correcting the piston corer depth based on magnetic susceptibility and X-ray fluorescence core scanner data. The upper 56 cm of the trigger corer complements the missing section of the piston corer yielding a composite depth for core PS97/085-3 (Supplementary Table 1).

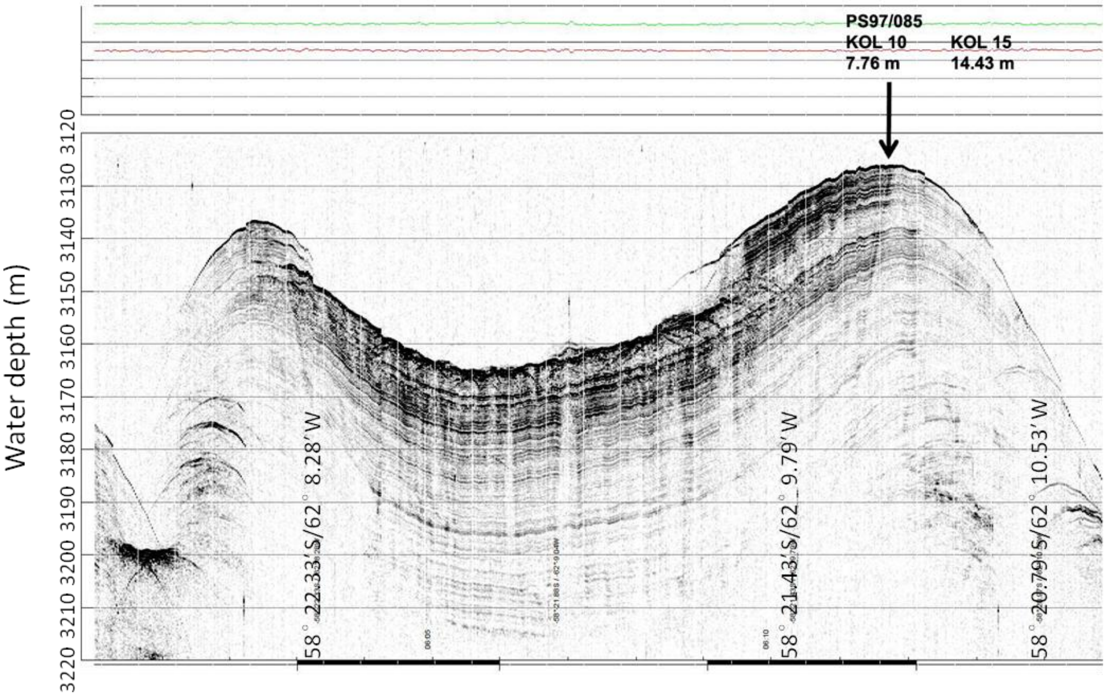

Supplementary Figure 1. Parasound profile across the Scotia Ridge east of the Shackleton Fracture Zone with the coring site PS97/085[1].

Core PS97/085-3 primarily consists of terrigenous material (mean 90.5% [weight %], min. 62.3%, max. 98.5%) and biogenic carbonate, mainly foraminifera shells in the coarse sand fraction. The terrigenous components are characterized by brownish fine sand to sandy silt during warm periods, whereas cold periods correspond to grayish silt to blue-gray silty clay[1]. Measurements of the total carbon (TC) and organic carbon (TOC) contents through element analyzes were used to calculate the $CaCO_3$ content by [$CaCO_3$ = (TC-TOC)* 8.333]. $CaCO_3$ contents range from ~20 to 35% during the interglacials and are less than 10% for the glacial periods. TOC contents vary from 0.05 to 0.18 wt % and biogenic opal content is very low (mean 2.2%, min. 0.8%, max. 5.1%) throughout the core.

Magnetic Susceptibility

The response function of the MS2E sensor with respect to a thin magnetic layer[2] is equivalent to a Gaussian curve with a half-width of slightly less than 4 mm. The amplitude resolution of the sensor is 10^{-5} (SI-Units) in combination with the MS2 unit. During data acquisition, after every 10th measurement on sediment, the sensor is lifted to about 4 cm above the sediment in order to take a blank reading in air. This is done in order to monitor the shift of the sensor's background due to temperature drift. Subsequently, the air readings were linearly interpolated and subtracted from the readings on sediment.

Paleomagnetic measurements

For paleo- and rock magnetic investigation core PS97/085-3 was sampled with clear plastic boxes with an internal size of 20×20×15 mm (6 cm^3) pressed side by side into the split core. The anisotropy of magnetic susceptibility (AMS) was determined on these boxes using an AGICO Multi-Function Kappabridge MFK-1S, applying a field of 200 mAm^{-1} at a frequency of 976 Hz. The orientations of the principle axes of the AMS ellipsoid, giving information about the status of the magnetic fabric, were provided by the AGICO software 'Safyr6'. The natural remanent magnetization (NRM) was measured and stepwise demagnetized with a superconducting 2G Enterprises 755 SRM long-core magnetometer, with in-line tri-axial alternating field demagnetizer, and an automated sample handler for 8 discrete samples. Demagnetization was accomplished in 10 steps at AF peak levels of 5, 10, 15, 20, 30, 40, 50, 65, 80, and 100 mT. Obtained NRM demagnetization results were analysed with principle component analysis (PCA) according to Kirschvink et al. (1980)[3], in order to determine the direction of the characteristic remanent magnetization (ChRM). An anhysteretic remanent magnetization (ARM) was imparted using a separate 2G Enterprises 600 AF demagnetizer equipped with an additional coil for a static field.

ARMs were stepwise demagnetized with a reduced number of steps: 10, 20, 30, 40, 50, 65, and 80 mT. The relative paleointensity (RPI) was determined by the slope of NRM/ARM during alternating field demagnetization.

X-ray fluorescence core scanning

The X-ray fluorescence core scanner (XRF-CS) provides non-destructive, high resolution, and semi-quantitative chemical composition records. The elements (Al to Fe) were measured with 10 kV and 150 µA (No-filter) for 10 s. The elements Br to Sr were measured with 30 kV and 175 µA (thick Pd-filter) for ~15 s and elements up to Ba were measured at 50 kV and 1000 µA (Cu-filter) for ~20 s, all per 1 cm step (1.2 cm width). The specific element peak area intensity of the fluorescence spectrum is proportional to the concentration of the corresponding element. To minimize sample inhomogeneity, variable water content and grain-size distribution effects, we based our interpretation on the log-ratios of element intensities. Weltje and Tjallingii (2008) suggested the log ratios are close to the element concentration ratios[4].

Age Models

We established the age model based on radiocarbon dates, paleomagnetic excursion, corrected RPI with the RPI stack[5] and fine tuning points from high resolution XRF-CS ln(Ca/Ti) (Supplementary Table 1). Planktonic foraminifera *Neogloboquadrina pachyderma* were selected for radiocarbon measurements, performed with the Mini Carbon Dating System (MICADAS) at the AWI, Bremerhaven. The Accelerator Mass Spectrometry ^{14}C ages were calibrated to calendar ages using the MARINE 20 calibration curve[6] and the local reservoir age of ΔR = 974 years[7]. The RPI from core PS97/085-3 was correlated with the paleomagnetic intensity stack from 16 sediment core records from the Black Sea[5]. The high-resolution XRF scanner-derived records of ln(Ca/Ti) (peak area count ratios) were applied to fine-tune to the Antarctic temperature record on the ice core chronology AICC2012 using the AnalySeries Software[8,9].

The high-resolution XRF scanner-derived records of ln(Ca/Ti) and ln(Ca/Fe) from PS97/085-3 show very similar pattern (Supplementary Fig. 3). The variations of Ti and Fe in relation to Ca indicate strong climate-related variations of biogenic carbonate (mainly foraminifers) to the terrigenous sediment fraction in this region. The carbonate content is primarily controlled by biogenic productivity and carbonate dissolution (lysocline), which is determined by the carbonate saturation state in the deep water[10,11]. The terrigenous sediment input varies through changing input from southern Patagonia and Antarctica. Both areas were strongly glaciated during glacial periods. We assigned 24 tuning points (Supplementary Table 1) from the ln(Ca/Ti)

record correlated to the Antarctic temperature anomaly throughout the entire core. High sedimentation and mass accumulation rates prevailed during the full glacial periods (20~40 cm ka^{-1}; 30~50 g cm^{-2} ka^{-1}; Supplementary Fig. 3).

Grain-size measurements

The detrital fraction of the sediments was isolated from the bulk sediment through removal of the organic matter by hydrogen peroxide (15%), the carbonates by hydrochloric acid (0.5 mol/l). Low content of biogenic opal (<5 wt%) was not deemed necessary to be removed. 1 ml (0.05 mol/l) sodium metaphosphate ($Na_4P_2O_7$*$10H_2O$) was used to to ensure disaggregation of all particles. Grain-size analyses were carried out with a CILAS 1180L laser diffraction particle-size analyzer (CILAS, Orleans, France) at the AWI, Sylt. The device provides a measured grain size range between 14.6 and -1.32 ϕ (phi scale) corresponding to 0.04 µm–2500 µm in 0.1 ϕ steps. The analysis error of replicate testing is less than 3%.

For the comparison of data gathered with different equipments, 80 samples were selected to be measured as well with a Micromeritics SediGraph 5100 at AWI, Bremerhaven (Supplementary Fig. 7). To separate the terrigenous sediment fraction, we used 35% (vol/vol) H_2O_2 to remove organic matter and for disaggregation and 10% (vol/vol) acetic acid to dissolve the carbonate. Because biogenic silica contents are low (1 to 5 wt%), we did not dissolve opal. The separation of the sand fraction (63–2000 µm) was achieved by wet sieving, whereas the silt (2–63 µm) and clay fractions (<2 µm) were split by Stokes' Law settling using Atterberg tubes. To separate the latter two size fractions almost completely, a 10- to 12-times repetition of the settling procedure was necessary. Coagulation of clay size particles was avoided by using a 0.1% sodium polyphosphate solution. Grain-size analyses of the silt fraction (2–63 µm) were performed by measurements with the SediGraph. The SediGraph analyses provide a high-resolution grain-size distribution in steps of 0.1 ϕ and are based on the X-ray density scanning of a settling suspension assuming Stokes' Law settling. The instrument precision of the SediGraph 5100 for pure standard analysis ranges from ±0.3% to ±1.9%[12].

Computer tomography

The sediment core PS97/085-3 was scanned by a Toshiba Aquilion 64™ computer tomography (CT) at the hospital Klinikum Bremen-Mitte (Bremen, Germany). The X-ray source voltage was 120 kV and the current was 600 mA. The CT scan resolution is 0.35 mm in the x-y direction and 0.5 mm in the z direction (0.3 mm reconstruction interval). The CT images were reconstructed by the Toshiba patented helical cone beam reconstruction technique (TCOT). The CT data were processed

with the Amira ZIB edition software (version 2015.37; http://amira.zib.de)[13]. All lithic clasts >1 mm and bioturbation traces were segmented in each reconstruction slice with the "Threshold" segmentation tool of the Segmentation Editor. The used density threshold values were >1500 (Hounsfield units) for lithic clasts, 601–1499 for matrix sediment, 1–600 for bioturbation traces, and <1 for the surrounding air and water. Each material was quantified with the "Material Statistics" module (Volume per slice). Subsequently, the lithic clasts were separated with the "Connected Components" module, parameterized with the "Shape Analysis" module and a grain-size and orientation analysis (relative to z as well as within the x-y plane) performed following the methodology from Titschack et al. (2015)[14]. Each analysis considered a core interval of ~1 cm and moved slice by slice. The result (unit: vol.% of all segmented clasts) was written to the central slice of the interval.

Clasts (>1 mm) from CT results were classified as ice-rafted debris (IRD). The volume percentages and the number of clasts from CT analysis need to be considered together for interpretation of precise IRD distribution (Supplementary Fig. 5). The IRD abundances evaluated by only the volume percentages would overestimate large clasts, whilst just the number of clasts might exaggerate the significance of small debris[15]. CT data show that relatively higher IRD abundances prevailed during the glacial periods, reaching the maximum during MIS 6 (2 vol.%; 25 clasts cm^{-3}). Generally, most of the IRD contents are lower than 2 vol.% except for a peak of 18 vol.% at ~60 ka. This spike might be caused by few large clasts, which is suggested by the low number of <3 clasts cm^{-3} (Supplementary Fig. 5). The IRD contents significantly decreased during the deglaciation and interglacial periods (min 0 vol.%, Supplementary Fig. 5).

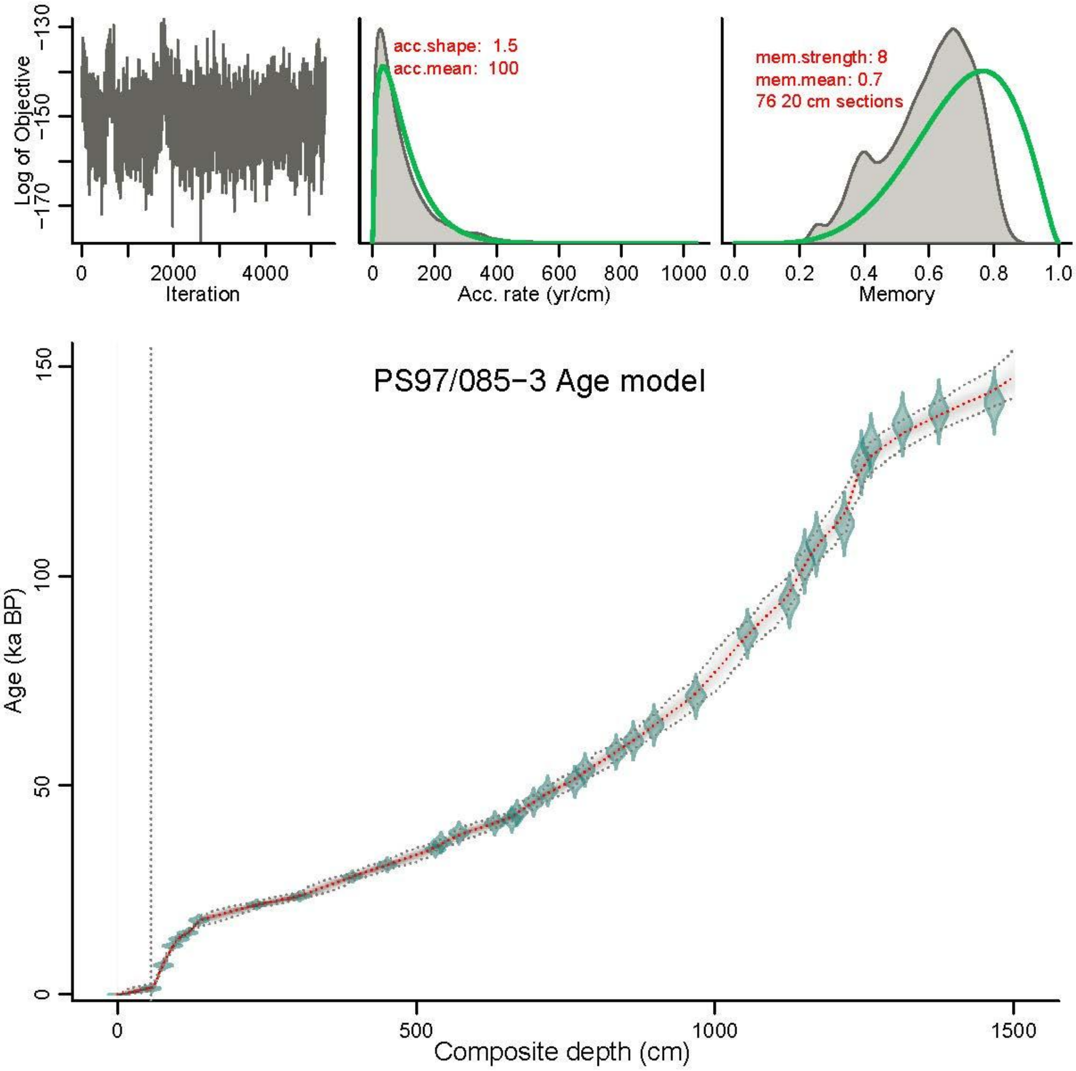

Supplementary Figure 2. Bayesian age-depth model of core PS97/085-3 calculated with R program BACON[16]. The vertical dashed line is the merging depth between trigger and piston cores showing as well possible coring artifacts.

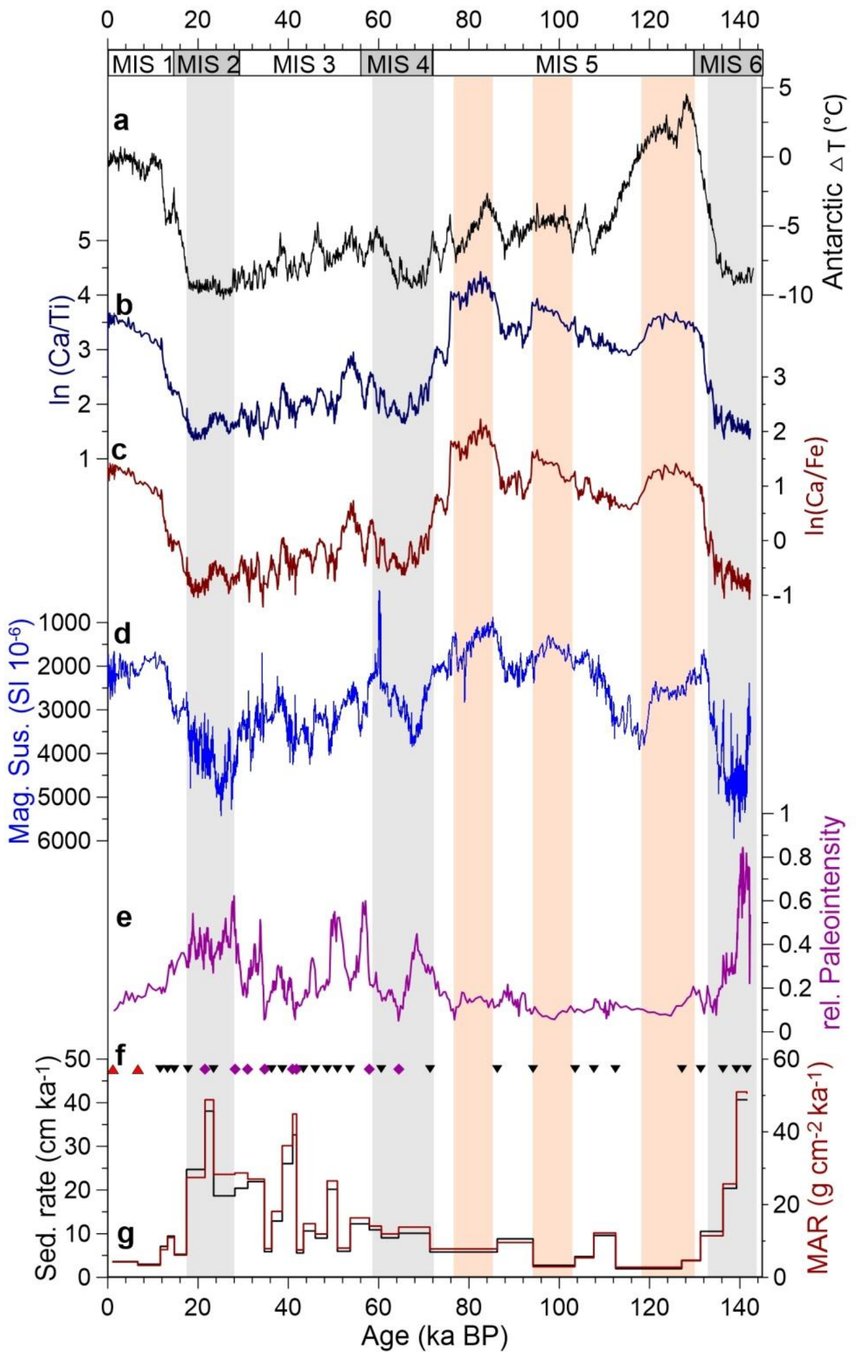

Supplementary Figure 3. Age model for sediment core PS97/085-3. Age control points shown in **f** are based on radiocarbon dates (red triangles) and the paleomagnetic excursions[17,18] (purple diamonds). Additional control points (black down triangles) are from (**b**) XRF-derived ln(Ca/Ti) profile of PS97/085-3 tuning with (**a**) the Antarctic temperature anomaly time series[9]. XRF-derived ln(Ca/Ti) is consistent with climate induced changes of the ln(Ca/Fe) in **b**, **c**. **d**, Volume magnetic susceptibility (Mag.Sus., inverted axis) record on core PS97/085-3. **e** Relative paleointensity variations (RPI, inverted axis) on core PS97/085-3. **g** Linear sedimentation rates (sed.rate, black) and mass accumulation rates (MAR, red). Vertical gray bars mark inferred glacial periods and pink bars mark sub-interglacials during Marine Isotope Stages (MIS) 5.

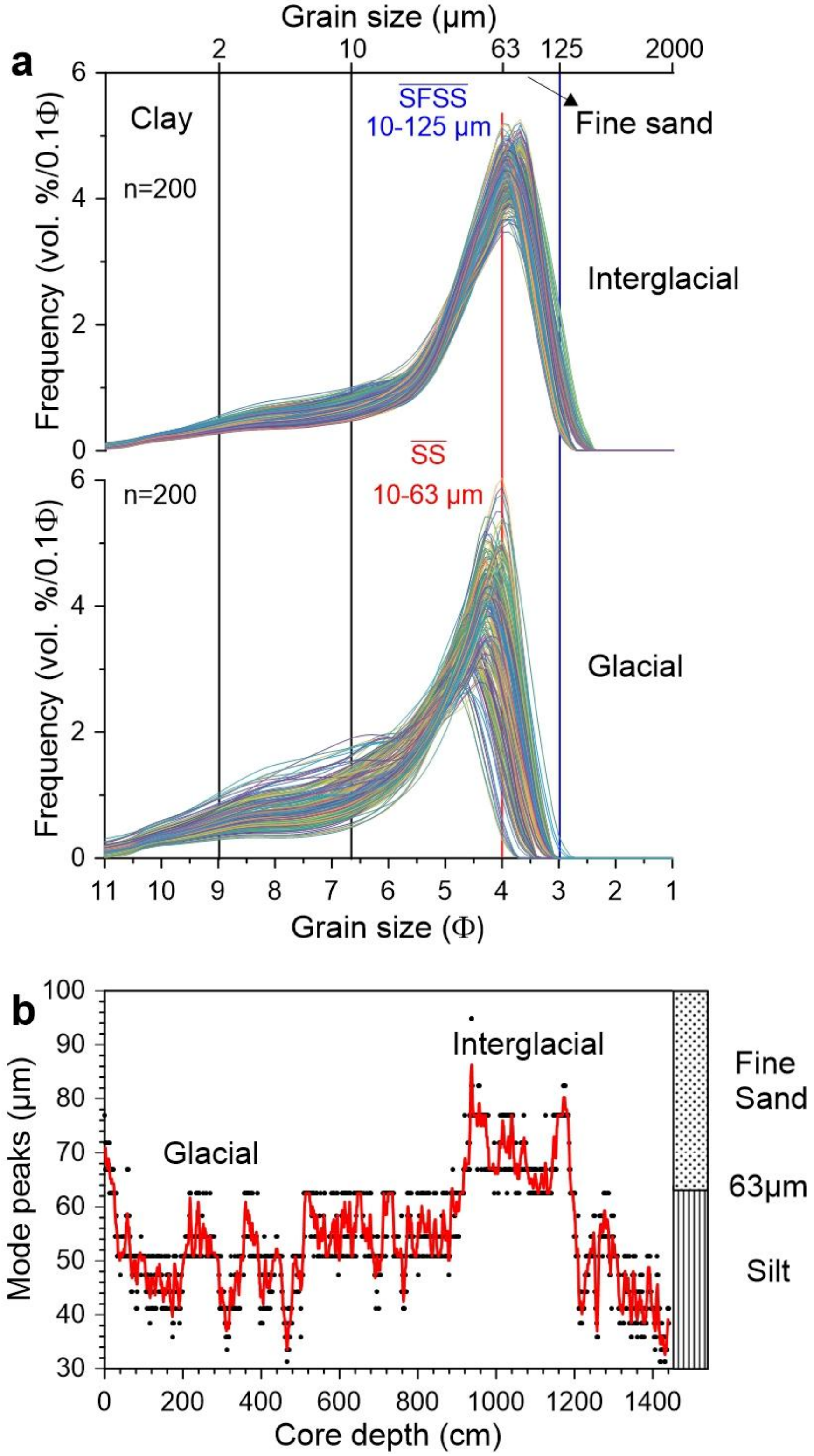

Supplementary Figure 4. The grain size distribution of terrigenous sediments from core PS97/085-3. **a** The mode of grain size distribution moved to coarser fractions under high flow speed during the interglacial compared to glacial period. The mean sortable silt and fine-sand ($\overline{\mathrm{SSFS}}$) is a range of $10-125$ μm compared with the mean sortable silt ($\overline{\mathrm{SS}}, 10-63$ μm). **b** Mode peaks shifted beyond the silt-sand boundary (63 μm) during interglacial but stayed below the boundary during the glacial periods.

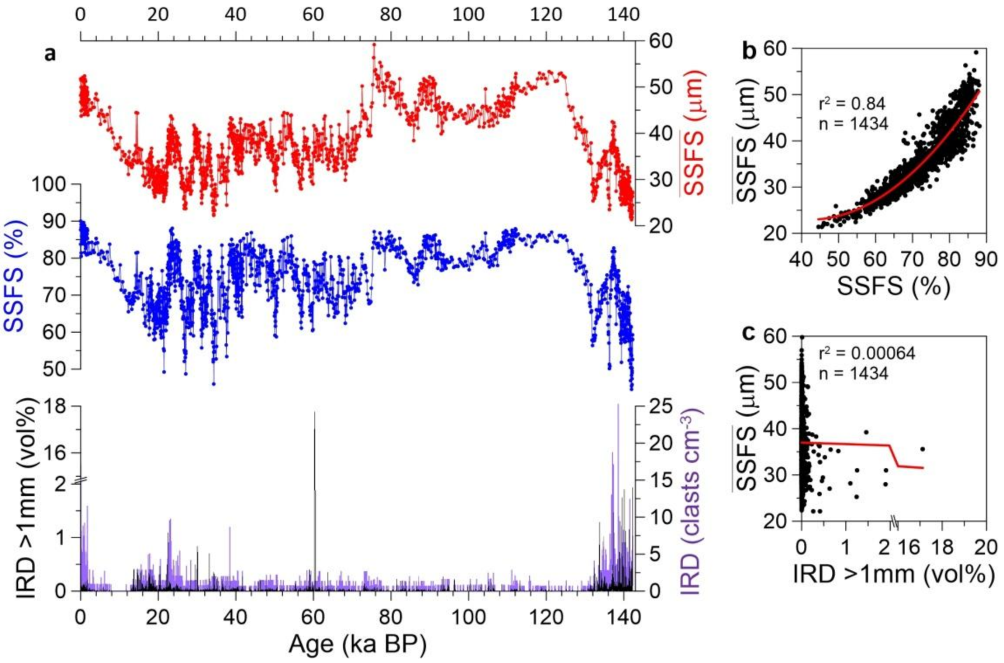

Supplementary Figure 5. Grain-size data and ice-rafted debris (IRD) contents from core PS97/085-3. **a** the variations of $\overline{SSFS}$, SSFS percentage and >1 mm fraction IRD (vol.% and number of clasts); **b** Polynomial regression between $\overline{SSFS}$ and SSFS; **c** $\overline{SSFS}$ and IRD are independent of each other.

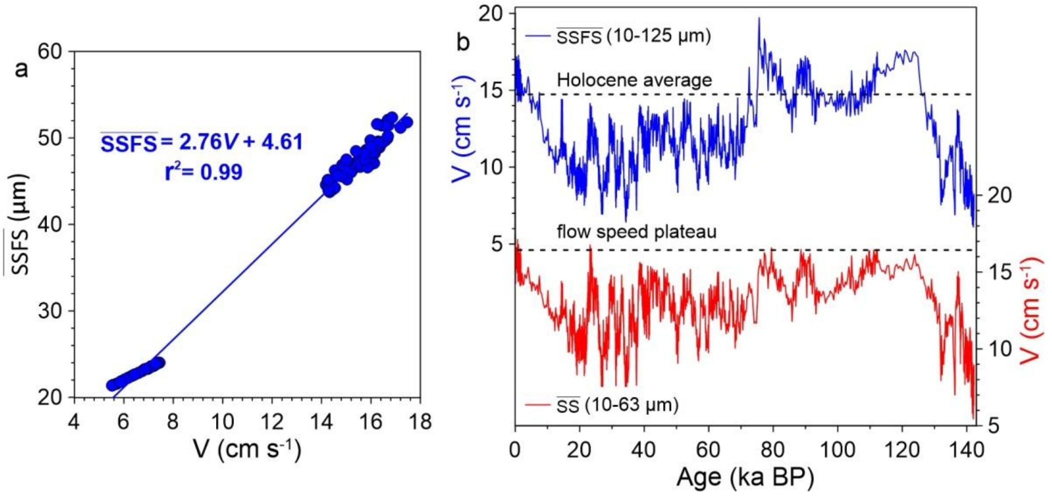

Supplementary Figure 6. a Calibration of mean grain size of the sortable silt plus fine sand fraction (blue) and the mean sortable silt (red). The modern current speed is ~15 cm s^{-1} from modern current meter C18[19] nearby our sediment core, in response to the geometric mean grain sizes during the last 4 ka ($\overline{SS} = \sim 35$ μm, $\overline{SSFS} = \sim 48$ μm). We assume that lowest flow speeds have an insignificant effect on the coarse fraction, because the weight percentage of 63-125 μm is close to zero. Thus, we applied the surface sample scalar flow speed (V) calibration ($\overline{SS} = 1.31V + 14.22$) to calculate the flow speed values from these fines grain sizes ($\overline{SS}$ < 24 μm; n=28) and the samples of the last 4 ka (n=60) from our $\overline{SS}$ record. We took these values to calculate the function between $\overline{SSFS}$ and the scalar flow speed ($\overline{SSFS} = 2.76V + 4.61$). **b** PS97/085-3 scalar flow speed fluctuations (cm s^{-1}) calculated from the equation of $\overline{SSFS}$ (blue) and $\overline{SS}$ (red). Apparently, the flow speeds reach its plateau reconstructed when calculated with $\overline{SS}$.

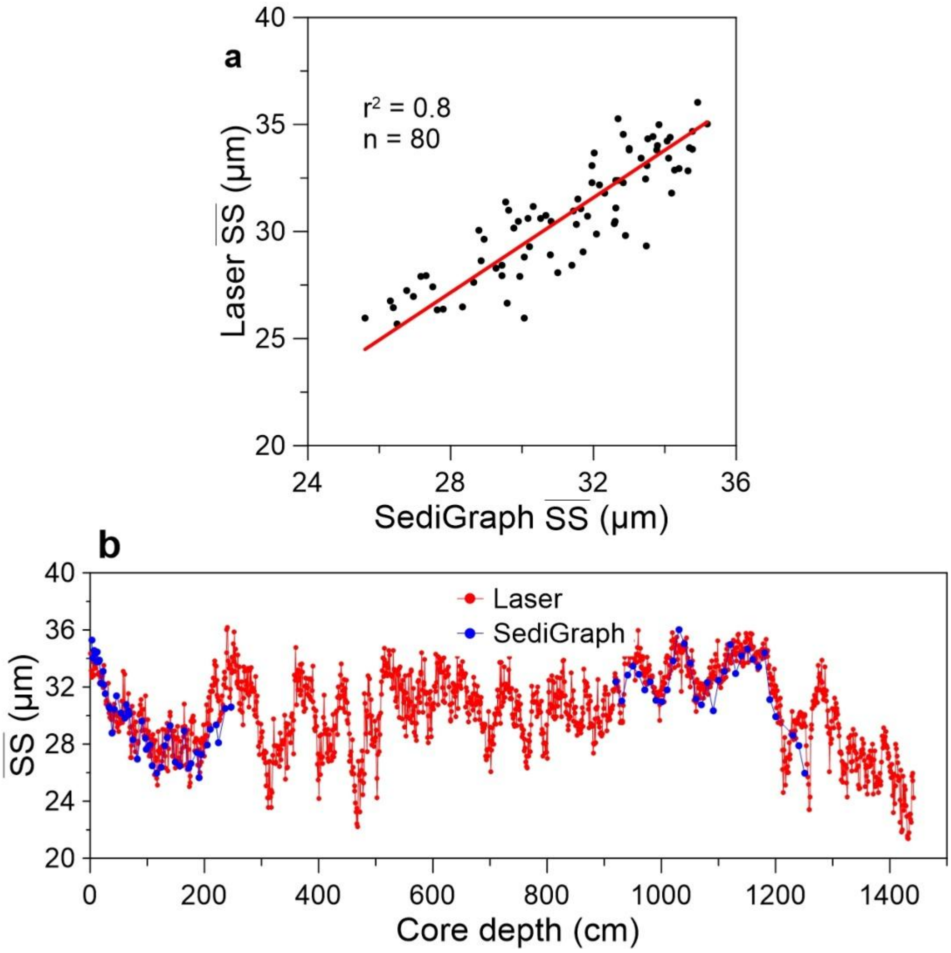

Supplementary Figure 7. Comparison of $\overline{SS}$ values analyzed with the SediGraph (blue) and CILAS laser measurements (red) in core PS97/085-3. **a** The similar results obtained by these different devices support the robustness of our grain-size measurement. **b** Absolute Laser $\overline{SS}$ values tend to be slightly coarser than the SediGraph data, because the sand fraction was separated before the SediGraph measurements and with that as well some of the coarse silt was removed, but amplitudes are generally consistent.

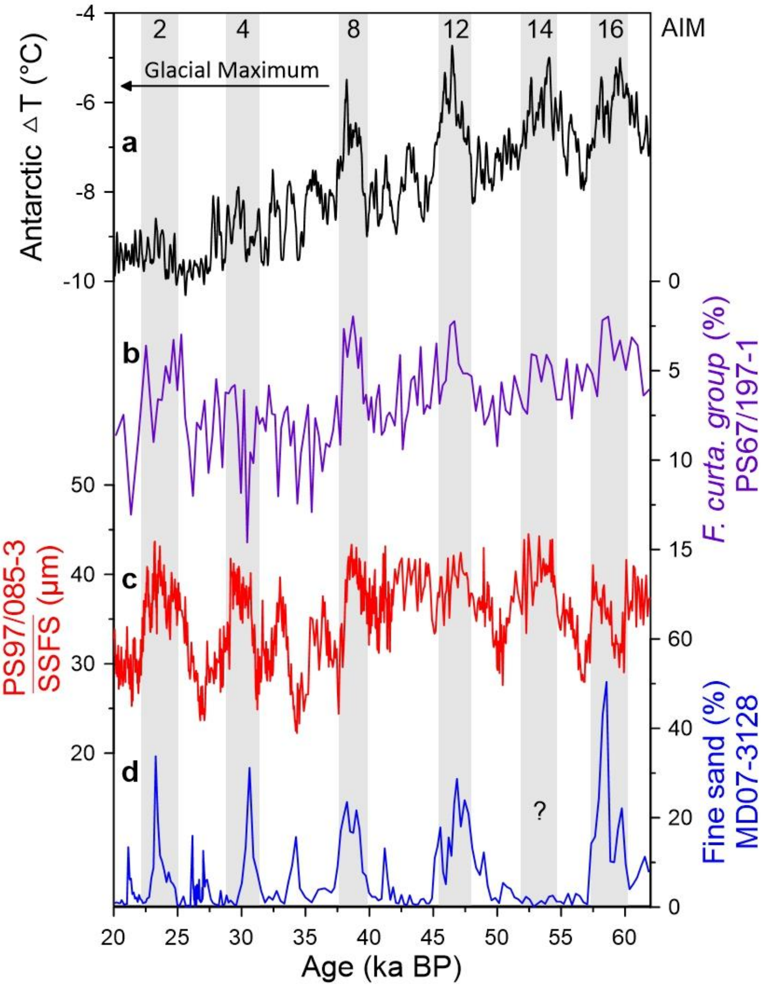

Supplementary Figure 8. Enhanced sensitivity of the millennial ACC variations to the Southern Hemisphere climate oscillation. **a** The Antarctic temperature anomaly declined to the minimum value during the glacial maximum[9]. **b** Diatom-based *Fragilariopsis curta* group (*F. curta* + *F. cylindrus*) indicates winter sea ice extent[20]. **c** and **d**, The proxies for the ACC flow speeds, $\overline{SSFS}$ from the central Drake Passage (this study) and fine sand percentage from sediment core MD07-3128 at the Chilean Margin[21], are corresponding to the Southern Hemisphere climate oscillation and increased sensitivity of the ACC under the glacial maximum climate background. AIM, Antarctic Isotope Maximum.

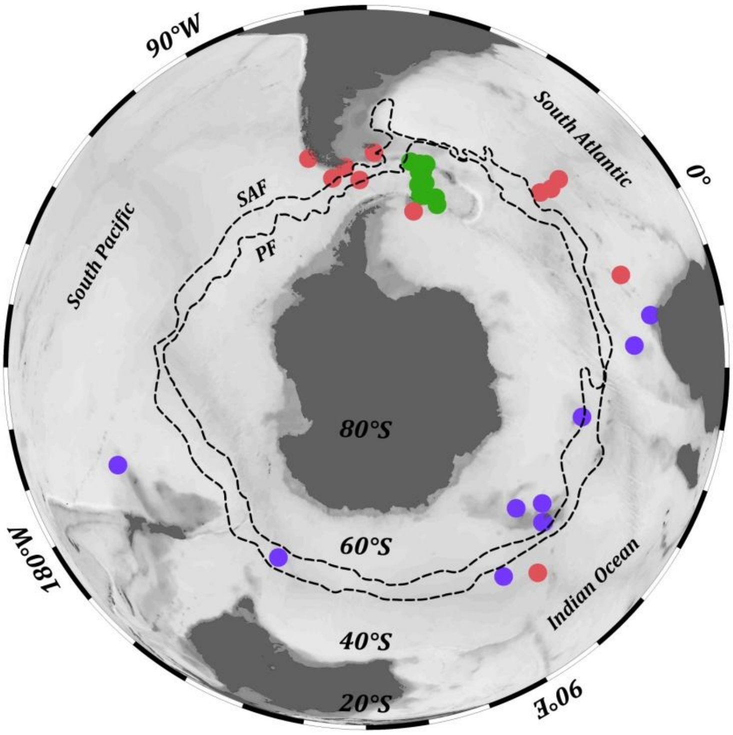

Supplementary Figure 9. Reconstructed glacial-interglacial changes in ACC flow speeds in the Southern Ocean. Red dots[21-23] mark sites where the reconstructed ACC strength increased during interglacials compared to glacials. Purple dots[24-29] indicate sites with an ACC strength decrease during interglacials compared to glacials. Green dots[30] mark sites with no significant changes in reconstructed ACC strength between interglacials and glacials. A consistent reduction in ACC strength during glacial is documented in the Drake Passage region north of the PF and an possible ACC-related reduction of bottom currents in the subantarctic South Altlantic sector[31-33]. Reconstructions of the ACC strength in the Indian Ocean sector reveal enhanced glacial flow speeds in the Antarctic Zone but show inconsistent pattern north into the subantarctic Southern Ocean[26-29,34]. Some bottom current reconstructions could be more related to dense bottom water flow (like modern Antarctic Bottom Water) especially in the deeper basins[31-33]. Note proxies are not always identical, like some of the reconstructed ACC strength in the Indian Ocean are based on density calculations[29] and magnetic grains[26].

Supplementary Table 1. Age control points for the construction of age model for core PS97/085-3.

Composite depth (cm)	Piston core depth (cm)	^{14}C age (ka)	±Error (ka)	Reservoir age (ka)	Calibrated age (ka)	±Error (ka)	Dating materials
56.5	0.5	2.70	0.11	0.97	1.13	0.27	Foraminifera
76.5	20.5	7.32	0.13	0.97	6.64	0.30	Foraminifera

Composite depth (cm)	Piston core depth (cm)	Age (ka BP)	±Error* (ka)	Dating method
0	0	0	0	Trigger core surface
56.5	0.5	1.13	0.27	^{14}C/MARINE 20
76.5	20.5	6.64	0.30	^{14}C/MARINE 20
91.5	35.5	11.64	0.24	ln(Ca/Ti) tuning
103.0	47.0	13.26	0.28	ln(Ca/Ti) tuning
117.0	61.0	14.74	0.24	ln(Ca/Ti) tuning
136.0	80.0	17.78	0.33	ln(Ca/Ti) tuning
232.5	176.5	21.55	0.31	Paleomagnetic
305.0	249.0	23.45	0.37	ln(Ca/Ti) tuning
393.0	337.0	28.16	0.46	Paleomagnetic
451.0	395.0	31.01	0.51	Paleomagnetic
532.0	476.0	34.70	0.68	Mono Lake
541.5	485.5	36.32	0.73	ln(Ca/Ti) tuning
571.5	515.5	38.64	0.78	ln(Ca/Ti) tuning
631.5	575.5	40.95	0.81	Laschamp
660.0	604.0	41.82	0.81	Laschamp
668.5	612.5	43.34	0.93	ln(Ca/Ti) tuning
696.5	640.5	45.98	0.98	ln(Ca/Ti) tuning
720.0	664.0	48.59	1.02	ln(Ca/Ti) tuning
765.5	709.5	50.85	1.07	ln(Ca/Ti) tuning
782.5	726.5	53.70	1.12	ln(Ca/Ti) tuning
834.5	778.5	57.94	1.25	Paleomagnetic
863.5	807.5	60.60	1.32	ln(Ca/Ti) tuning
898.0	842.0	64.42	1.44	Norwegian-Greenland Sea
968.0	912.0	71.35	1.59	ln(Ca/Ti) tuning
1054.5	998.5	86.26	1.78	ln(Ca/Ti) tuning
1124.5	1068.5	94.20	2.07	ln(Ca/Ti) tuning
1150.0	1094.0	103.46	2.07	ln(Ca/Ti) tuning
1170.0	1114.0	107.65	2.07	ln(Ca/Ti) tuning
1216.5	1160.5	112.49	2.07	ln(Ca/Ti) tuning
1245.5	1189.5	127.11	2.07	ln(Ca/Ti) tuning
1261.5	1205.5	131.28	2.07	ln(Ca/Ti) tuning
1314.0	1258.0	136.27	2.08	ln(Ca/Ti) tuning
1375.0	1319.0	139.26	2.07	ln(Ca/Ti) tuning
1467.5	1411.5	141.54	2.07	ln(Ca/Ti) tuning

* The error estimate for tuning points and paleomagnetic points was using mean squared estimate following the method suggested by Grant et al. (2012)[35].

Supplementary Table 2. ACC flow speeds reconstruction and comparison across the Drake Passage.

Core. no	Projected on Track 104[36] (km)	IG	$\overline{SS}$ (µm)	$\overline{SSFS}$ (µm)	SEM	IG V (cm s^{-1})	G	$\overline{SS}$ (µm)	$\overline{SSFS}$ (µm)	SEM	G V (cm s^{-1})	G-IG V differences (cm s^{-1})	G-IG V changes (% from IG)
1. MD07-3128[21]	0	Hol	38.3	n/a	2.54	18.4	LGM	21.4	n/a	0.69	5.5	-12.9	-70
2. GC528[23]	20	Hol	30.2	n/a	1.09	12.2	LGM	20.1	n/a	0.53	4.5	-7.7	-63
3. MR0806-PC9[21]	50	Hol	33.9	n/a	2.68	15.0	LGM	27.3	n/a	0.38	10.0	-5.0	-33
4. PS97/093-2[22]	70	Hol	25.6	n/a	0.43	8.7	LGM	23.1	n/a	0.18	6.8	-1.9	-22
		MIS 5e	25.2	n/a	0.45	8.4	PGM	22.3	n/a	0.35	6.2	-2.2	-26
SAF	80	n/a	n/a	n/a	n/a	n/a	n/a	n/a	n/a	n/a	n/a	n/a	n/a
SSAF	280	n/a	n/a	n/a	n/a	n/a	n/a	n/a	n/a	n/a	n/a	n/a	n/a
5. PS97/085-3	420	Hol	32.2	n/a	0.43	13.7	LGM	29.3	n/a	0.33	11.5	-2.2*	-16*
		MIS 5e	33.6	n/a	0.40	10.0	PGM	25.7	n/a	0.39	6.9	-3.1*	-31*
(this study)		Hol	n/a	45.3	1.01	14.6	LGM	n/a	32.4	0.55	9.7	-4.9	-34
		MIS 5e	n/a	48.2	1.77	15.7	PGM	n/a	26.9	0.57	7.6	-8.2	-52
APF	465	n/a	n/a	n/a	n/a	n/a	n/a	n/a	n/a	n/a	n/a	n/a	n/a
6. TPC063[30]	470	Hol	21.4	n/a	0.60	5.5	LGM	19.8	n/a	0.62	4.2	-1.2	-22
7. TPC077[30]	490	Hol	19.6	n/a	0.81	4.1	LGM	20.3	n/a	0.60	4.6	+0.5	12
8. PS2514-1[30]	505	Hol	19.0	n/a	0.46	3.6	LGM	19.6	n/a	0.62	4.1	+0.5	14
9. PS67/197-1[30]	538	Hol	17.4	n/a	0.63	2.4	LGM	17.7	n/a	0.50	2.7	+0.3	13
10. TPC078[30]	590	Hol	16.1	n/a	0.33	1.4	LGM	16.9	n/a	0.28	2.0	+0.6	43
11. PS67/205-2[30]	715	Hol	18.1	n/a	0.56	2.9	LGM	16.2	n/a	0.26	1.5	-1.4	-48
12. PS67/219-1[30]	780	Hol	17.8	n/a	0.24	2.8	LGM	16.6	n/a	0.54	1.8	-1.0	-36
SACCF	780	n/a	n/a	n/a	n/a	n/a	n/a	n/a	n/a	n/a	n/a	n/a	n/a
13. PS67/224-1[30]	806	Hol	16.0	n/a	0.26	1.4	LGM	15.5	n/a	0.32	1.0	-0.4	-29
14. TPC288[30]	840	Hol	17.5	n/a	0.21	2.5	LGM	16.7	n/a	0.25	1.9	-0.6	-24
15. PS67/186-1[30]	870	Hol	15.2	n/a	0.33	0.7	LGM	14.9	n/a	0.24	0.5	-0.2	-29
16. PS2319-1[30]	890	Hol	15.8	n/a	0.48	1.1	LGM	15.5	n/a	0.52	0.9	-0.2	-18
17. TPC287[30]	920	Hol	15.6	n/a	0.24	1.1	LGM	16.0	n/a	0.31	1.3	+0.3	26

Note that SEM is standard error of mean grain-size ($2\sigma/\sqrt{n}$). Holocene (0-12 ka); LGM (18-28 ka); MIS 5e (116-129 ka); PGM (~140 ka). SAF, Subantarctic Front; SSAF, southern SAF; APF, Antarctic Polar Front; SACCF, southern ACC Front. G, Glacial; IG, Interglacial. *$\overline{SS}$ underestimates the amplitude of the ACC changes, not taken for mean V change calculation. n/a, not applicable.

Supplementary References

1 Lamy, F. The Expedition PS97 of the Research Vessel POLARSTERN to the Drake Passage in 2016. *Berichte zur Polar-und Meeresforschung Reports on polar and marine research* **701**, (2016).

2 Nowaczyk, N. *Logging of magnetic susceptibility in Tracking environmental change using lake sediments*. Vol. 1: Basin analysis, coring, and chronological techniques. 155–170 (Kluwer Academic Publishers, 2001).

3 Kirschvink, J. L. The least-squares line and plane and the analysis of palaeomagnetic data. *Geophysical Journal International* **62**, 699-718, (1980).

4 Weltje, G. J. & Tjallingii, R. Calibration of XRF core scanners for quantitative geochemical logging of sediment cores: Theory and application. *Earth Planet Sc Lett* **274**, 423-438, (2008).

5 Liu, J., Nowaczyk, N. R., Panovska, S., Korte, M. & Arz, H. W. The Norwegian-Greenland Sea, the Laschamps and the Mono Lake excursions recorded in a Black Sea sedimentary sequence spanning from 68.9 to 14.5 ka. *Journal of Geophysical Research: Solid Earth*, (2020).

6 Heaton, T. J. *et al.* MARINE20—THE MARINE RADIOCARBON AGE CALIBRATION CURVE (0–55,000 CAL BP). *Radiocarbon*, 1-42, (2020).

7 Chen, T. *et al.* Synchronous centennial abrupt events in the ocean and atmosphere during the last deglaciation. *Science* **349**, 1537-1541, (2015).

8 Paillard, D., Labeyrie, L. & Yiou, P. Macintosh Program performs time-series analysis. *Eos, Transactions American Geophysical Union* **77**, 379-379, (1996).

9 Jouzel, J. *et al.* Orbital and millennial Antarctic climate variability over the past 800,000 years. *Science* **317**, 793-796, (2007).

10 Bohm, E. *et al.* Strong and deep Atlantic meridional overturning circulation during the last glacial cycle. *Nature* **517**, 73-76, (2015).

11 Sigman, D. M., Hain, M. P. & Haug, G. H. The polar ocean and glacial cycles in atmospheric CO(2) concentration. *Nature* **466**, 47-55, (2010).

12 Bianchi, G., Hall, I. R., McCave, I. & Joseph, L. Measurement of the sortable silt current speed proxy using the Sedigraph 5100 and Coulter Multisizer IIe: Precision and accuracy. *Sedimentology* **46**, 1001-1014, (1999).

13 Stalling, D., Westerhoff, M. & Hege, H.-C. Amira: A highly interactive system for visual data analysis. *The visualization handbook* **38**, 749-767, (2005).

14 Titschack, J. *et al.* Aggradation and carbonate accumulation of Holocene Norwegian cold-water coral reefs. *Sedimentology* **62**, 1873-1898, (2015).

15 Bartels, M. *et al.* Atlantic Water advection vs. glacier dynamics in northern Spitsbergen since early deglaciation. *Clim. Past* **13**, 1717-1749, (2017).

16 Blaauw, M. & Christen, J. A. Flexible paleoclimate age-depth models using an autoregressive gamma process. *Bayesian analysis* **6**, 457-474, (2011).

17 Benson, L. *et al.* Age of the Mono Lake excursion and associated tephra. *Quaternary Sci Rev* **22**, 135-140, (2003).

18 Nowaczyk, N. R., Arz, H. W., Frank, U., Kind, J. & Plessen, B. Dynamics of the Laschamp geomagnetic excursion from Black Sea sediments. *Earth Planet Sc Lett* **351-352**, 54-69, (2012).

19 Donohue, K., Tracey, K., Watts, D., Chidichimo, M. & Chereskin, T. Mean Antarctic Circumpolar Current transport measured in Drake Passage. *Geophys Res Lett* **43**, (2016).

20 Xiao, W., Esper, O. & Gersonde, R. Last Glacial - Holocene climate variability in the Atlantic sector of the Southern Ocean. *Quaternary Sci Rev* **135**, 115-137, (2016).

21 Lamy, F. *et al.* Glacial reduction and millennial-scale variations in Drake Passage throughflow. *Proceedings of the National Academy of Sciences* **112**, 13496-13501, (2015).

22 Toyos, M. H. *et al.* Antarctic Circumpolar Current dynamics at the Pacific entrance to the Drake Passage over the past 1.3 million years. *Paleoceanography and Paleoclimatology* **35**, e2019PA003773, (2020).

23 Roberts, J. *et al.* Deglacial changes in flow and frontal structure through the Drake Passage. *Earth Planet Sc Lett* **474**, 397-408, (2017).

24 Beny, F. *et al.* Radiogenic isotopic and clay mineralogical signatures of terrigenous particles as water-mass tracers: New insights into South Atlantic deep circulation during the last termination. *Quaternary Sci Rev* **228**, 106089, (2020).

25 Hall, I. R., McCave, I. N., Shackleton, N. J., Weedon, G. P. & Harris, S. E. Intensified deep Pacfic inflow and ventilation in Pleistocene glacial times. *Nature* **412**, 809-812, (2001).

26 Mazaud, A., Michel, E., Dewilde, F. & Turon, J. Variations of the Antarctic Circumpolar Current intensity during the past 500 ka. *Geochemistry, Geophysics, Geosystems* **11**, (2010).

27 Molyneux, E. G., Hall, I. R., Zahn, R. & Diz, P. Deep water variability on the southern Agulhas Plateau: Interhemispheric links over the past 170 ka. *Paleoceanography* **22**, (2007).

28 Martínez-Méndez, G., Zahn, R., Hall, I. R., Pena, L. D. & Cacho, I. 345,000-year-long multi-proxy records off South Africa document variable contributions of Northern versus Southern Component Water to the Deep South Atlantic. *Earth Planet Sc Lett* **267**, 309-321, (2008).

29 Lynch-Stieglitz, J., Ito, T. & Michel, E. Antarctic density stratification and the strength of the circumpolar current during the Last Glacial Maximum. *Paleoceanography* **31**, 539-552, (2016).

30 McCave, I., Crowhurst, S., Kuhn, G., Hillenbrand, C. & Meredith, M. Minimal change in Antarctic Circumpolar Current flow speed between the last glacial and Holocene. *Nat Geosci* **7**, 113-116, (2014).

31 Diekmann, B. *et al.* In *The South Atlantic in the Late Quaternary* Ch. 4 (Springer, Berlin, 2003).

32 Diekmann, B. & Kuhn, G. Terrigene Partikeltransporte als Abbild spätquartärer Tiefen- und Bodenwasserzirkulation im Südatlantik und angrenzendem Südpolarmeer. *Zeitschrift der Deutschen Geologischen Gesellschaft:* **148**, 405-429, (1997).

33 Kuhn, G. & Diekmann, B. Late Quaternary variability of ocean circulation in the southeastern South Atlantic inferred from the terrigenous sediment record of a drift deposit in the southern Cape Basin (ODP Site 1089). *Palaeogeogr Palaeocl* **182**, 287-303, (2002).

34 Williams, T. J. *et al.* Neodymium isotope evidence for coupled Southern Ocean circulation and Antarctic climate throughout the last 118,000 years. *Quaternary Sci Rev* **260**, 106915, (2021).

35 Grant, K. M. *et al.* Rapid coupling between ice volume and polar temperature over the past 150,000 years. *Nature* **491**, 744-747, (2012).

36 Koenig, Z., Provost, C., Ferrari, R., Sennéchael, N. & Rio, M.-H. Volume transport of the

Antarctic Circumpolar Current: Production and validation of a 20 year long time series obtained from in situ and satellite observations. *Journal of Geophysical Research: Oceans* **119**, 5407-5433, (2014).
